# FAMILIAL AND SOCIODEMOGRAPHIC DETERMINANTS OF PLACE OF DEATH: A RETROSPECTIVE STUDY OF THE UTAH POPULATION DATABASE

**DOI:** 10.1093/geroni/igac059.1415

**Published:** 2022-12-20

**Authors:** Brenna Kelly, Heidi Hanson, Huong Meeks, Michael Hollingshaus, Djin Tay, Lee Ellington, Caroline Stephens, Katherine Ornstein

**Affiliations:** University of Utah, Salt Lake City, Utah, United States; University of Utah, Salt Lake City, Utah, United States; University of Utah, Salt Lake City, Utah, United States; University of Utah, Salt Lake City, Utah, United States; University of Utah, Salt Lake City, Utah, United States; University of Utah and Huntsman Cancer Institute, Salt Lake City, Utah, United States; University of Utah, Salt Lake City, Utah, United States; Johns Hopkins, University, Baltimore, Maryland, United States

## Abstract

Consistent with preferences, home deaths in the US increasing — yet most Americans still die in hospitals or other healthcare facilities. Although declining health has been considered the primary factor influencing place of death, few studies have examined how family support and sociodemographic factors influence place of death. This study examined a population-based cohort of 205,932 decedents aged 50+ who died in Utah between 1998 and 2016. Using multivariate logistic regression models, we found that having a living spouse or child was associated with decreased odds of a healthcare facility death (spouse: AOR= 0.62, CI 0.65-0.59; child: AOR = 0.80, CI 0.79-0.82). Additionally, educational attainment (graduate degree: AOR = 0.95, CI 0.91-0.99) and non-Hispanic/Latinx ethnicity (AOR = 0.81, CI 0.79-0.85) were associated with decreased odds of a home death. Our findings highlight the importance of families in place of death and suggest that sociodemographic and economic disparities persist even in death.

